# Synergistic Effects and Mechanisms of Combined Treatment With Harmine Hydrochloride and Azoles for Resistant *Candida albicans*

**DOI:** 10.3389/fmicb.2019.02295

**Published:** 2019-10-15

**Authors:** Xiuyun Li, Xuexin Wu, Yan Gao, Lina Hao

**Affiliations:** Department of Pharmacy, Qilu Children’s Hospital, Shandong University, Jinan, China

**Keywords:** harmine hydrochloride, azoles, resistant *Candida albicans*, *Galleria mellonella*, synergistic mechanism

## Abstract

Several studies have demonstrated the significant antiviral, antimicrobial, antiplasmodial, antioxidative, antifungal, antimutagenic, and antitumor properties of harmine hydrochloride (HMH). The main objective of the present study was to investigate the antifungal effects and underlying mechanisms of HMH when combined with azoles to determine whether such combinations act in a synergistic manner. As a result, we found that HMH exhibits synergistic antifungal effects in combination with azoles against resistant *Candida albicans* (*C. albicans*) planktonic cells, as well as resistant *C. albicans* biofilm in the early stage. Antifungal potential of HMH with fluconazole was also explored *in vivo* using an invertebrate model. Our results suggest that HMH combined with azoles is synergistic against resistant *C. albicans in vitro* and *in vivo*. No synergy is seen with azole sensitive *C. albicans* strains nor with other *Candida* species. Such synergistic mechanisms on resistance *C. albicans* are involved in increasing intracellular azoles, inhibiting hyphal growth, disturbing cytosolic calcium concentration, and inducing apoptosis of resistant *C. albicans* cells.

## Introduction

Despite therapeutic advances, candidiasis remains the most common fungal infection, 50% of which is caused by *Candida albicans* (*C. albicans*) (Yapar, 2014; Pu et al., 2017). *C. albicans* can usually be associated with high mortality (Wisplinghoff et al., 2004). The choice of appropriate antifungals is very important for patients with the *C. albicans* infection treatment. Therapies aimed to treat these infections are limited to azoles, polyenes, and echinocandins. Among them, azoles are the most commonly used antifungals due to their better efficacy and higher safety levels. Increases in resistant *C. albicans* complicate the therapy for *C. albicans* infections. Therefore, managing resistant fungi and discovering drug alternatives against resistant *C. albicans* are significant challenges for clinical therapy and antifungal drug development. Importantly, understanding the inner mechanisms associated with fungal resistance is particularly important for overcoming drug resistance and developing new antifungal agents. In recent years, the concept of combination antifungal therapy has become a research hotspot (Marr, 2004; Liu et al., 2014). Different drugs or compounds in combination with antifungal are recommended to inhibit *C. albicans*, especially resistant *C. albicans*.

Harmine hydrochloride (HMH) is an abundant alkaloid in nature and several studies have demonstrated its multiple pharmacological activities, such as antiviral, antibacterial, antiplasmodial, antioxidative, antifungal, antimutagenic, and anticancer properties (Zaker et al., 2007; Egusa et al., 2011; Frost et al., 2011; Hamsa and Kuttan, 2011; Yonezawa et al., 2011; Onishi et al., 2012; Reus et al., 2012; Chen et al., 2015). Although reports have been published on the antifungal effects of HMH (Patel et al., 2012), its synergistic effect with antifungal agents is yet to be reported.

Therefore, our aim was to determine the antifungal potential of HMH in combination with azoles *in vitro* and *in vivo*. Furthermore, we aimed to elucidate the antifungal mechanisms of these drug combinations. In this study, the microdilution method was used to determine the *in vitro* antifungal effects of HMH in combination with azoles on planktonic cells and biofilms. *G. mellonella* infection model was used to evaluate the *in vitro* antifungal effects of these drug combinations. In addition, changes in the biofilm formation, hyphal growth, cytosolic calcium concentration ([Ca^2+^]_i_) and apoptosis markers were measured to elucidate the anti-resistance mechanisms of these drug combinations.

## Materials and Methods

### Isolates and Media

As shown in Table 1, 12 *Candida* spp. isolates were used in this study. These isolates and the quality control isolate, ATCC 10231, were obtained from Shandong Provincial Qianfoshan Hospital (Jinan, China). Before each experiment, each isolate was revived twice on Sabouraud’s Dextrose Agar (SDA) solid medium for 24 h. RPMI (Roswell Park Memorial Institute) 1640 medium was used in the experiments.

**TABLE 1:** Drug interactions of HMH and azoles against twelve *Candida* spp. *in vitro.*

**Drugs**	**Isolates^a^**	**MIC_80_(μg/mL)^b^**				**FICI model^c^**	
		**MIC_azole_**	**C_azole_**	**MIC_HMH_**	**C_HMH_**	**FICI**	**Interpretation**
HMH + FLC	CA4 (S)	>0.5	0.125	>102	1024	1.250	No interaction
	CA8 (S)	0.5	0.25	>1024	1024	1.500	No interaction
	CA10 (R)	>512	0.25	512	8	0.016	**Synergism**
	CA16 (R)	>512	0.5	512	8	0.017	**Synergism**
	CG1 (S)	4	4	128	128	2.000	No interaction
	CG2 (R)	128	128	> 1024	>1024	2.000	No interaction
	CG3 (R)	32	32	>1024	>1024	2.000	No interaction
	CG8 (S)	8	8	128	128	2.000	No interaction
	CK2 (S)	4	4	128	128	2.000	No interaction
	CK3 (S)	8	4	128	128	1.500	No interaction
	CK9 (R)	32	32	>1024	>1024	2.000	No interaction
	CK10 (R)	128	128	>1024	>1024	2.000	No interaction
HMH + ITR	CA4 (S)	0.25	0.25	>1024	>1024	2.000	No interaction
	CA8 (S)	0.25	0.25	>1024	>1024	2.000	No interaction
	CA10 (R)	> 64	0.125	>1024	16	0.018	**Synergism**
	CA16 (R)	> 64	0.5	>1024	32	0.039	**Synergism**
	CG1 (S)	0.25	0.25	>1024	>1024	2.000	No interaction
	CG2 (R)	128	4	>1024	>1024	1.031	No interaction
	CG3 (R)	128	8	>1024	>1024	1.063	No interaction
	CG8 (S)	1	0.25	>1024	>1024	1.250	No interaction
	CK2 (S)	1	0.125	>1024	>1024	1.125	No interaction
	CK3 (S)	1	1	>1024	>1024	2.000	No interaction
	CK9 (R)	128	32	>1024	>1024	1.250	No interaction
	CK10 (R)	128	> 32	>1024	>1024	1.250	No interaction
HMH + VRC	CA4 (S)	0.25	0.25	>1024	>1024	2.000	No interaction
	CA8 (S)	0.25	0.25	>1024	>1024	2.000	No interaction
	CA10 (R)	32	0.0625	>1024	32	0.033	**Synergism**
	CA16 (R)	32	0.0625	>1024	32	0.033	**Synergism**
	CG1 (S)	0.25	0.25	>1024	>1024	2.000	No interaction
	CG2 (R)	4	4	> 1024	>1024	2.000	No interaction
	CG3 (R)	2	2	>1024	>1024	2.000	No interaction
	CG8 (S)	1	1	>1024	>1024	2.000	No interaction
	CK2 (S)	0.5	0.5	>1024	>1024	2.000	No interaction
	CK3 (S)	0.5	0.0313	>1024	>1024	1.063	No interaction
	CK9 (R)	2	2	>1024	>1024	2.000	No interaction
	CK10 (R)	4	4	>1024	>1024	2.000	No interaction

*^*a*^CA, *C. albicans*; CG, *Candida glabrata*; CK, *Candida krusei*; S, susceptible; R, resistant. ^*b*^MIC: minimum inhibitory concentration; MIC_*azole*_ and MIC_*HMH*_: the MICs when drugs used alone; C_*zaole*_ and C_*HMH*_: the MICs when drugs used in combination. ^*c*^ FICI ≤ 0.5: synergism; FICI > 4.0: antagonism; 0.5 < FICI ≤ 4.0: no interaction.*

### Antifungal Agents and *G. mellonella* Larvae

All reagents in this study were purchased from Dingguo Changsheng Biotech Co., Ltd. (Beijing, China). All stock solutions, including HMH (5,120 μg/mL in sterile distilled water), fluconazole (FLC, 2,560 μg/mL in sterile distilled water), itraconazole (ITR, 2,560 μg/mL in DMSO), voriconazole (VRC, 2,560 μg/mL in DMSO) and oxacillin sodium (2,560 μg/mL in sterile distilled water), were stored at −20°C. *G. mellonella* larvae were purchased from Tianjin Huiyude Biotech Co., Ltd. (Tianjin, China). Weighing (0.25 ± 0.03 g) of larvae was performed in all assays.

### Determination of Minimum Inhibitory Concentrations (MICs) of Planktonic Cells *in vitr*o

Minimum inhibitory concentrations of antifungal agents were tested based on CLSI standard reference method (M27-A3), using 96-well microtiter plates. The concentrations of azoles and HMH were adjusted to 0.125 μg/mL to 64 μg/mL, and 16 μg/mL to 1,024 μg/mL, respectively. Meanwhile, *Candida* spp. cell suspensions with a final concentration of 1.0 × 10^3^ cells/mL were added to the 96-well microtiter plates. Plates containing FLC were incubated for 24 h and plates containing ITR or VRC were incubated for 48 h at 35°C. After incubation, XTT assay was used for the detection of cell growth at 492 nm by the microplate reader. MIC endpoints were read as previously described (Pfaller et al., 2008; Li et al., 2017). Experiments were performed in triplicate. The MIC of the control strain ATCC10231 was within the prescript of CLSI M27-A3.

### Determination of Sessile Minimum Inhibitory Concentrations (SMICs) of Biofilms *in vitr*o

*Candida albicans* biofilm formation was performed as described elsewhere with some modifications (Vila et al., 2013; Wang et al., 2015). The biofilms of CA10 and CA16 were formed over 90 min or 24 h at 35°C by adding 200 μL of the standardized cell suspension (5.0 × 10^6^ cells/mL) into selected wells of 96-well plates. After 90 min or 24 h, the wells were gently washed three times with sterile PBS to remove any remaining non-adhering cells. HMH and azoles were then added to the wells. After incubation for 48 h at 35°C, XTT assay was used for detection at 492 nm by the microplate reader. The drug concentration that reduces the absorption value by 50% relative to control was regarded as the SMIC endpoint. Experiments were performed in triplicate.

### Drug Interaction Interpretation

Interactions of the drug combination were interpreted according to the fractional inhibitory concentration index (FICI) model based on Loewe Additivity (LA) theory as previously described (Odds, 2003; Li et al., 2017).

### Determination of *in vivo* Antifungal Effects

#### Survival Assay

*Galleria mellonella* survival assays were carried out according to the previously described method with few modifications (Mylonakis et al., 2005; Scorzoni et al., 2013). The larvae weighing (0.25 ± 0.03 g) were employed in all assays. A resistant isolate, CA10, was used in this experiment. Larvae were stored in wood chips without light at 25°C before the start of the experiments. Each group contained 20 randomly chosen larvae. *C. albicans* cells were incubated in liquid SDA medium for 24 h. Cell suspensions of *C. albicans* 5 × 10^8^ cells/mL were prepared in PBS and 20 μg/mL of oxacillin sodium was added to prevent bacterial infection. Larvae were inoculated in *C. albicans* suspensions (final inoculated concentration: 5 × 10^6^ cells/larva) as previously described (Li et al., 2017). At 2.5 h after post-infection, infected larvae were treated with FLC (final concentration *in vivo*: 1.6 μg/larva) and HMH (final concentration *in vivo*: 1.6 μg/larva) alone or in combination. A group of infected larvae without drug treatment was regarded as control. Larvae were kept on petri dishes at 35°C and monitored daily for survival. Death was identified when there was no movement after touching with metal tweezers. All assays were repeated on three separate occasions.

#### Histological Study

*Galleria mellonella* larvae were treated with CA10 and drugs as described above. Three days after incubation, one larva from each group was collected, washed and fixed in 4% paraformaldehyde for 24 h. Subsequently, larvae were embedded using optimal cutting temperature compound and 8-μm tissue slices of larvae were made using a freezing microtome. Each tissue slice was processed as previously described (Li et al., 2017). All assays were repeated on three separate occasions.

### Synergistic Mechanism Explorations

#### Assay for the Uptake and Efflux of Rhodamine 6G (R6G)

R6G was used to specifically detect whether HMH affects the intracellular concentration of azoles in resistant *C. albicans* isolate (CA10). The experiment was conducted as previously described (Li et al., 2017), with minor modifications. The *C. albicans* cells were incubated by shaking at 180 rpm in SDA liquid medium at 35°C for 18 h and harvested at mid-log phase by centrifugation at 3000 rpm and 4°C for 10 min. The samples were washed twice with PBS and re-suspended in PBS.

For the R6G uptake assay, R6G (10 μM) and HMH (128 μg/mL) were simultaneously added to the prepared cells. Cells treated with R6G alone were regarded as control. The BD FACSAria II flow cytometer (excitation at 488 nm, emission at 530 nm) was used to determine mean fluorescent intensity (MFI) every 10 min. Each assay was performed in triplicate.

For the R6G efflux assay, cells were prepared in the same manner described above and starved for 2 h at 35°C. The starved cells were harvested at 3000 rpm and 4°C for 10 min and resuspended in PBS. Subsequently, 1.0 × 10^7^ cells/mL was incubated in the presence of R6G (10 μM) in 5 mL PBS at 35°C for 60 min to preload the cells with the R6G. Preloaded cells were harvested and treated in an ice bath for 15 min to stop preloading. Cells were pelleted by centrifugation at 3000 rpm and 4°C for 10 min, washed twice with PBS, and resuspended in PBS. Finally, HMH (128 μg/mL) was added to 5% glucose-PBS. Another group without HMH treatment was regarded as control. The MFI was determined with a flow cytometer every 10 min. Each assay was performed in triplicate.

#### Hypha Growth Assay

Two hundred μL suspension of CA10 cells (1.0 × 10^5^ cells/mL) treated with FLC (1 μg/mL) and HMH (32 μg/mL) alone or in combination was incubated in 96-well microtiter plates at 35°C for 4 h. Sample without drug treatment was regarded as control. Plates were shaken at 80 rpm for cell attachment to the surfaces of the disks. Unattached cells were subsequently removed, and the disks were washed twice with PBS. All samples were observed under a Leica DMI3000 microscope with the 10× objective and the 10× eyepiece. The experiment was performed in triplicate.

#### Cytosolic Calcium Concentration ([Ca^2+^]i) Measurement

The fluorescent probe Fluo-3/AM (3 mM) was used to determine the changes of [Ca^2+^]_i_ with different drug treatment. The drug free control was set with only Fluo-3/AM. The intensity of Fluo-3/AM fluorescence in *C. albicans* cells was measured in detail as R6G above and calculated with the following formula:

[Ca]2+=iK×d(F-F)min/(F-maxF)

where *K*_d_ is the calcium dissociation constant of the Fluo-3/AM combination (204 nmol/L at 37°C), *F* is the MFI of each sample, and *F*_min_ and *F*_max_ are the minimum and maximum fluorescence intensity of the measured values, respectively. The excitation and emission wavelengths for flow cytometer (Becton Dickinson, United States) were 488 and 530 nm, respectively. The fluorescence intensity of each sample was measured every 10 min until 60 min. Each experiment was performed three times.

#### ROS Production Assay

*Candida albicans* cells in the exponential phase were collected and washed thrice with PBS. thrice, cells (1 × 10^8^ cells/mL) were treated or untreated with HMH (32 μg/mL) and/or FLC (1 μg/mL) for 3 h. After incubation, cells were added to 2, 7-dichlorofluorescin diacetate (DCFH-DA, Sigma, United States) and incubated for 30 min in the dark. Before the detection, cells were washed with PBS for three times to remove the residual DCFH-DA in darkness conditions. MFI values were instantly detected on the BD FACSAria II flow cytometer (excitation at 488 nm, emission at 530 nm).

#### Metacaspase Activity Detection

Detection of activated metacaspases was performed using the CaspACE, FITC-VAD-FMK *In Situ* Marker (Promega, Madison, WI, United States). Briefly, CA10 cells treated with HMH (32 μg/mL) and/or FLC (1 μg/mL) for 12 h were collected, washed in PBS, and incubated with 10 μM of FITC-VAD- FMK for 1 h at 30°C in the dark. After incubation, cells were washed once and resuspended in PBS. Microscope and image acquisitions were performed with a Leica DMi8 fluorescence microscope.

### Statistics

Survival curves of *G. mellonella* were analyzed by the IBM SPSS statistics v.20.0 (IBM SPSS Inc., Chicago, IL, United States). Graphs of R6G and [Ca^2+^]_i_ were are presented as with Graph Pad Prism 7 (GraphPad Software Inc., California, CA, United States). *P* < 0.05 was considered statistically significant.

## Results

### Planktonic Cell Assay

The effects of HMH and azoles on *Candida* spp. were interpreted using the FICI model as shown in Table 1 and Supplementary Table S1. HMH exerted antifungal effects against six strains (CA10, CA16, CG1, CG8, CK2, and CK3) when used alone at 24 h. For the remaining six strains (CA4, CA8, CG2, CG3, CK9, and CK10) no antifungal effects were found when HMH was used alone. For resistant *C. albicans* strains (CA10, CA16), evident synergism of HMH in combination with three azoles was found. With 8 μg/mL of HMH, the MIC of FLC was reduced 2048-fold and 1024-fold for CA10 and CA16, respectively. With 16 or 32 μg/mL of HMH, the MIC of ITR was reduced 512-fold and 128-fold for CA10 and CA16, respectively. Besides, the MIC of VRC was reduced by 512-fold for both CA10 and CA16 with 32 μg/mL of HMH. No synergism was however observed for azole-sensitive *C. albicans* strains and azole resistant or sensitive strains of other *Candida* species.

### Biofilms Assay

The antibiofilm activity of HMH combined with three azoles on resistant *C. albicans* (CA10 and CA16) was interpreted by FICI. The antibiofilm results at 90 min and 24 h are shown in Tables 2, 3, respectively. Synergies were observed for the HMH and azole combinations against 90 min biofilms (Table 2), all with FICI < 0.5. However, no synergism was observed on the 24 h biofilms as shown in Table 3.

**TABLE 2:** Synergistic effects of HMH alone and in combination with azoles against 90 min biofilms of resistant *C. albicans.*

**Drugs**	**Isolates**	**SMIC_50_(μg/mL)^a^**				**FICI model^b^**	
		**SMIC_azole_**	**C_azole_**	**SMIC_HMH_**	**C_HMH_**	**FICI**	**Interpretation**
HMH + FLC	CA10	>128	0.25	>1024	16	0.018	**Synergism**
	CA16	>128	0.25	>1024	16	0.018	**Synergism**
HMH + ITR	CA10	>64	0.125	>1024	64	0.064	**Synergism**
	CA16	>64	0.25	>1024	64	0.066	**Synergism**
HMH + VRC	CA10	>64	0.125	>1024	64	0.064	**Synergism**
	CA16	>64	0.125	>1024	64	0.064	**Synergism**

*^*a*^SMIC: sessile minimum inhibitory concentration; SMIC_*azole*_ and SMIC_*HMH*_: the SMICs when drugs used alone; C_*azole*_ and C_*HMH*_: the SMICs when drugs used in combination. ^*b*^FICI ≤ 0.5: synergism; FICI > 4.0: antagonism; 0.5 < FICI ≤ 4.0: no interaction.*

**TABLE 3:** Synergistic effects of HMH alone and in combination with azoles against 24 h biofilms of resistant *C. albicans.*

**Drugs**	**Isolates**	**SMIC(μg/mL)^a^**				**FICI model^b^**	
		**SMIC_azole_**	**C_azole_**	**SMIC_HMH_**	**C_HMH_**	**FICI**	**Interpretation**
HMH + FLC	CA10	>128	>128	>1024	>1024	2.000	No interaction
	CA16	>128	>128	>1024	>1024	2.000	No interaction
HMH + ITR	CA10	>64	>64	>1024	>1024	2.000	No interaction
	CA16	>64	>64	>1024	>1024	2.000	No interaction
HMH + VRC	CA10	>64	>64	>1024	>1024	2.000	No interaction
	CA16	>64	>64	>1024	>1024	2.000	No interaction

*^*a*^SMIC: sessile minimum inhibitory concentration; SMIC_*azole*_ and SMIC_*HMH*_: the SMICs when drugs used alone; C_*azole*_ and C_*HMH*_: the SMICs when drugs used in combination. ^*b*^FICI ≤ 0.5: synergism; FICI > 4.0: antagonism; 0.5 < FICI ≤ 4.0: no interaction.*

### Survival Rate and Tissue Damage Assay

**FIGURE 1:**
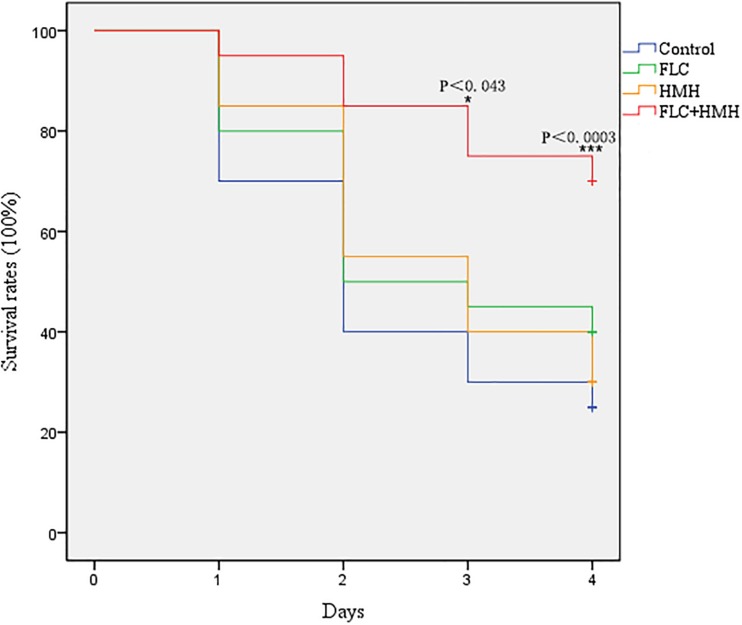
Effect of HMH alone or in combination with FLC on the survival rates of infected *G. mellonella.* The concentration of CA10 cells was 5 × 10^6^ CFU/larva. Treatments consisted of PBS, FLC (1.6 μg/larva) alone, HMH (1.6 μg/larva) alone, or a combination of FLC (1.6 μg/larva) with HMH (1.6 μg/larva). The IBM SPSS statistics v.20.0 software was used to analyze the data.

In the *in vivo* experiment, the resistant isolate CA10 was used, and FLC was selected as the representative of the three azoles. First, we determined whether this drug combination had an influence on the survival rates of infected *G. mellonella*. The combination of HMH and FLC leads to prolonged survival compared to the drug alone or control group. The difference in survival rates was statistically significant between the combination group and the other three groups (Figure 1), indicating the potential *in vivo* efficacy of this drug combination on resistant *C. albicans*.

**FIGURE 2:**
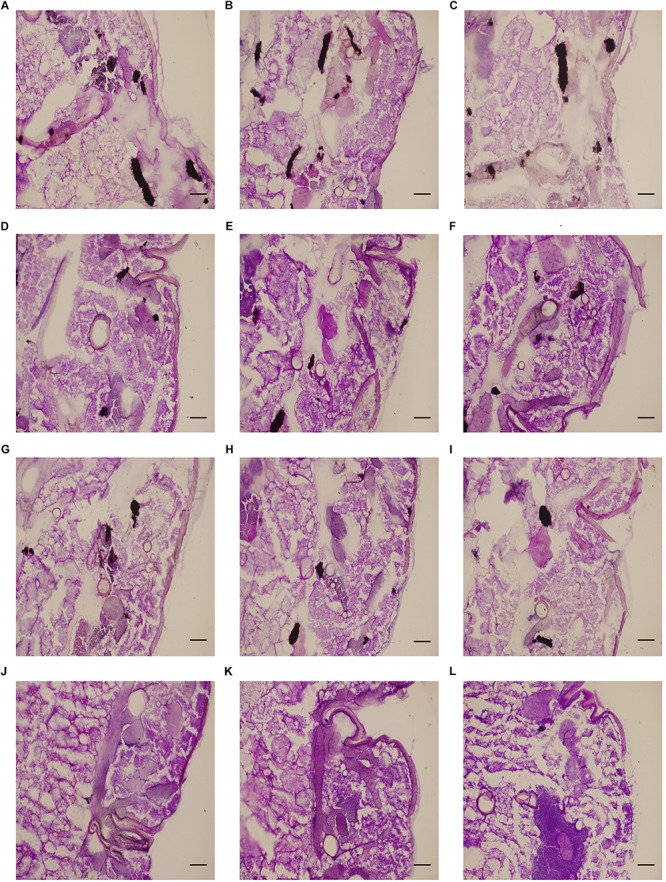
Effect of HMH alone or in combination with FLC on the histopathology of infected *G. mellonella.* The concentration of CA10 cells was 5 × 10^6^ CFU/larva. Treatments consisted of PBS **(A–C)**, FLC (1.6 μg/larva) alone **(D–F)**, HMH (1.6 μg/larva) alone **(G–I)**, or a combination **(J–L)** of FLC (1.6 μg/larva) with HMH (1.6 μg/larva). Melanized nodules consisted of mixtures of yeast cells and filaments. Bar = 200 μm.

Microscopic analysis was performed to determine the figures of infected *G. mellonella* tissues. In the control and drug monotherapy groups, abundant melanized nodules were observed in the tissues, which consisted of hyphae and yeast cells of *C. albicans*. However, the larvae that received drug combination treatment had a significant decrease in the number of melanized nodules compared to larvae in the other three groups (Figure 2).

### Uptake and Efflux of R6G Assay

**FIGURE 3:**
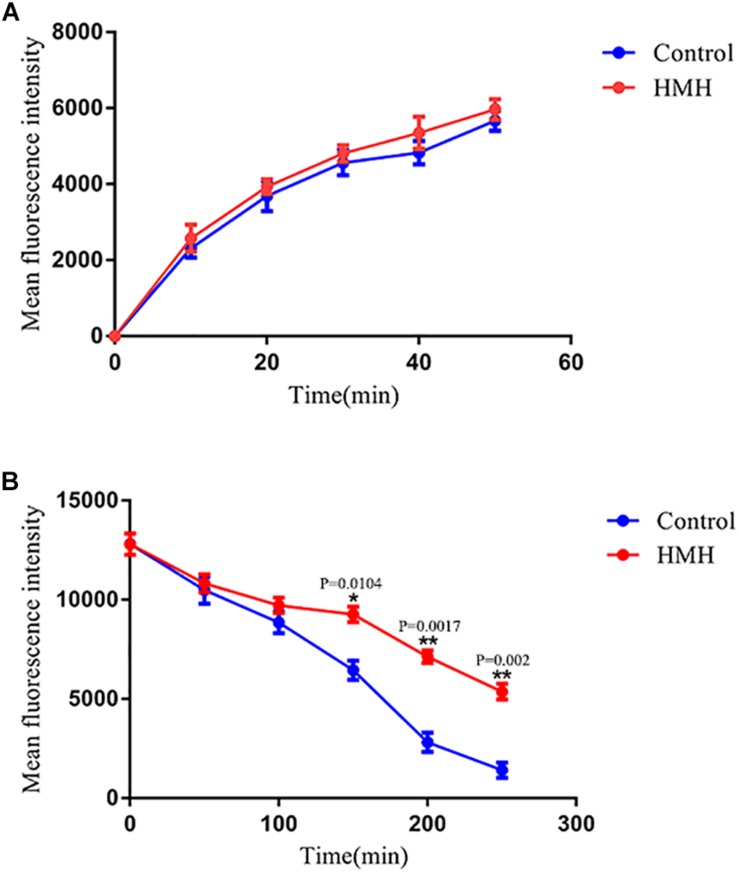
Effects of HMH on the **(A)** uptake and **(B)** efflux of R6G. The uptake and efflux of Rh6G in the absence and presence of HMH (128 μg/mL) were determined by a flow cytometer. Ten thousand events were counted for each sample at specific time intervals. MFIs represent the intracellular Rh6G in *C. albicans*. GraphPad Prism 7 software was used to analyze the data.

To clarify the influence of HMH on the uptake and efflux of azoles, resistant isolate CA10 was used. R6G was selected as the alternative for azoles. No difference was found in the intracellular R6G uptake of HMH-treated group and control group (Figure 3A). As shown in Figure 3B, the MFI in the HMH group was significantly higher than that in the control group at 150 min (*P* < 0.05), 200 min (*P* < 0.01), and 250 min (*P* < 0.01), indicating that HMH can inhibit the efflux of R6G in resistant *C. albicans*.

### Hyphal Growth Observation

**FIGURE 4:**
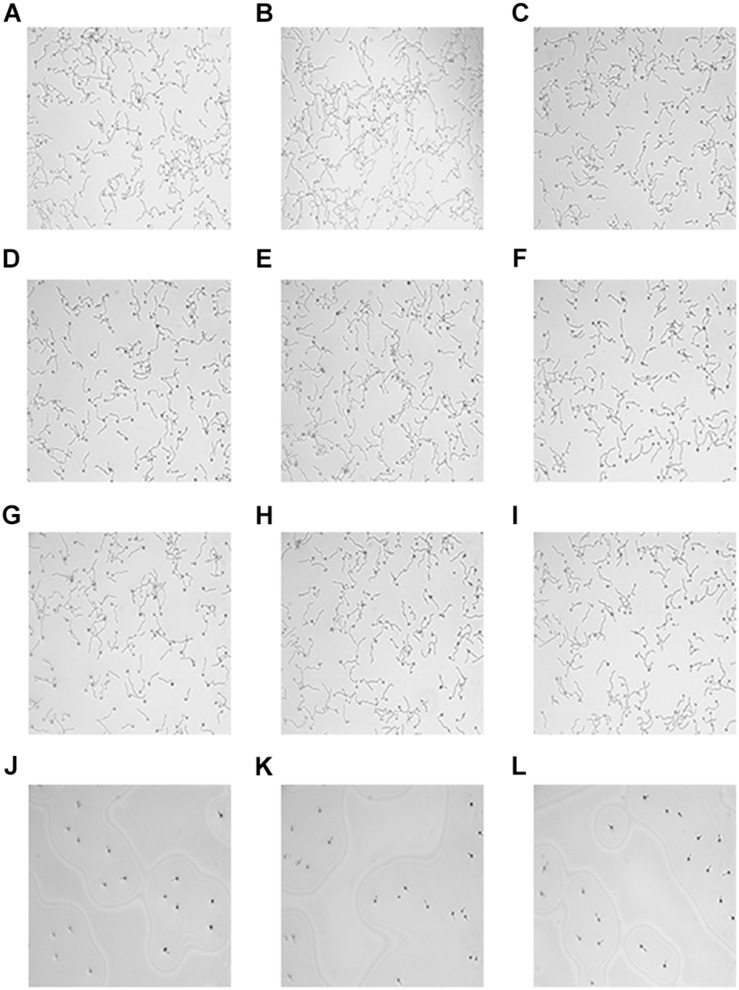
Microscope images hyphae. The concentration of CA10 cells was 1.0 × 10^5^ cells/mL. Drug treatments consisted of PBS **(A–C)**, FLC (1 μg/mL) alone **(D–F)**, HMH (32 μg/mL) alone **(G–I)**, and a combination **(J–L)** of FLC (1 μg/mL) with HMH (32 μg/mL).

Microscopic images were visualized to determine the effects of HMH and FLC against resistant *C. albicans* (CA10) hyphal growth. As illustrated in Figure 4, the control group had many biofilms with abundant filamentous growth and yeast forms. The same morphology was observed in the FLC group and HMH alone group while little hyphae were observed in the drug combination group. Based on the microscopic images illustrated in Figure 4, this drug combination was found to exert an inhibitory effect on hyphal growth of resistant *C. albicans*.

### [Ca^2+^]_i_ Measurement

**FIGURE 5:**
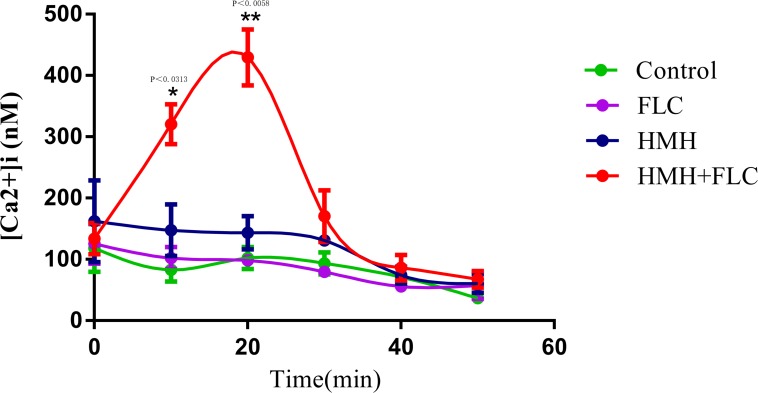
Changes of [Ca^2+^]i in resistant *C. albicans.* CA10 cells with Fluo-3/AM staining were analyzed using the flow cytometry. Drug treatments consisted of PBS, FLC (1 μg/mL), HMH (32 μg/mL), and a combination of FLC (1 μg/mL) with HMH (32 μg/mL). GraphPad Prism 7 software was used to analyze the data.

[Ca^2+^]_i_ was calculated using the aforementioned formula and the changes in [Ca^2+^]_i_ for each sample at different time points are shown in Figure 5. The results demonstrated that the drug combination group showed a significant increase in [Ca^2+^]_i_ at 10 and 20 min, indicating that the disorder of [Ca^2+^]_i_ may be one of the exact antifungal mechanisms for this drug combination against resistant *C. albicans* (CA10).

### ROS Production Assay

**FIGURE 6:**
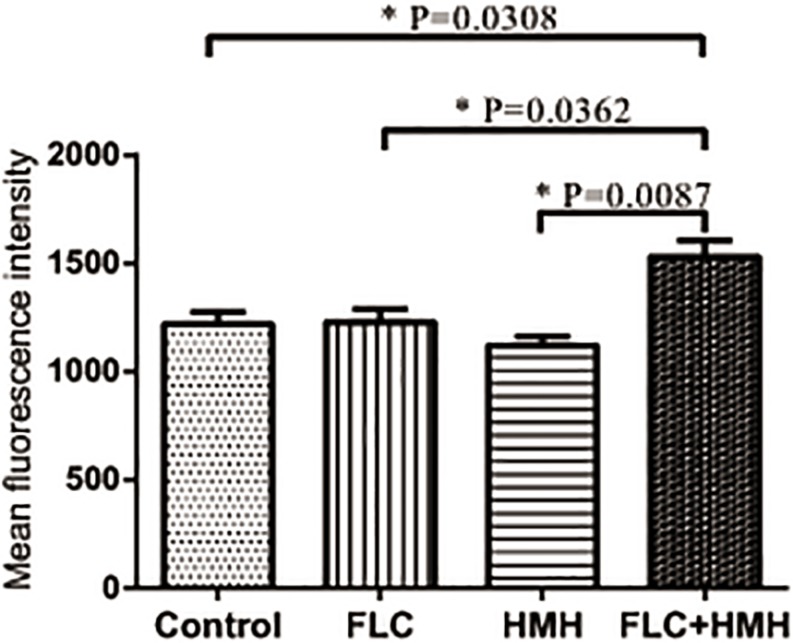
Effect of HMH alone or in combination with FLC on the production of ROS. CA10 cells with DCFH-DA staining were analyzed using the flow cytometry. Drug treatments consisted of PBS, FLC (1 μg/mL), HMH (32 μg/mL), and a combination of FLC (1 μg/mL) with HMH (32 μg/mL). GraphPad Prism 7 software was used to analyze the data.

To determine whether ROS participates in the synergistic mechanisms of HMH and FLC, the fluorescent probe DCFH-DA was used to monitor the production of intracellular ROS of resistant *C. albicans* (CA10) cells. Expectedly, cellular ROS level in the drug combination group was significantly higher than that in the other three groups (*P* < 0.05, Figure 6). This finding suggests that the remarkable accumulation of intracellular ROS partially results in synergistic antifungal effects of HMH and FLC against resistant *C. albicans*.

### Metacaspase Activity Detection

**FIGURE 7:**
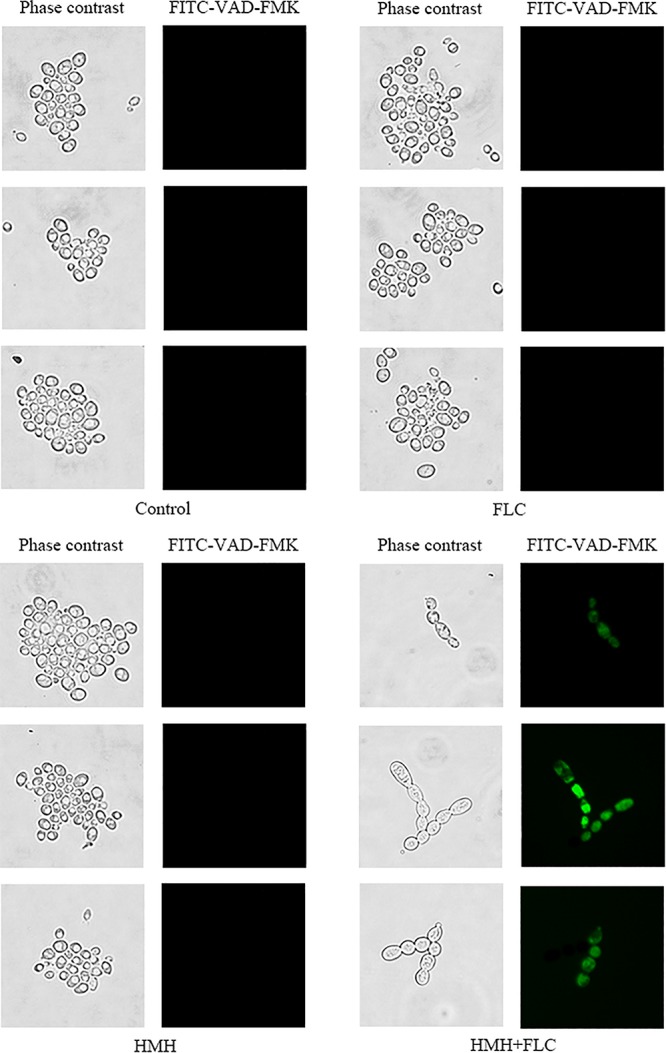
Effect of HMH alone or in combination with FLC on metacaspase activity. CA10 cells were collected, stained with FITC-VAD-FMK, and observed under a fluorescent microscope. Drug treatments consisted of PBS, FLC (1 μg/mL), HMH (32 μg/mL), and a combination of FLC (1 μg/mL) with HMH (32 μg/mL).

CaspACE FITC-VAD-FMK is a fluorescent dye that binds specifically to the active site of caspases. Although caspases are not present in fungi, the orthologs of caspases in animals, called metacaspases, have been identified in fungi and plants, and their activity can be assessed using the same fluorescent dye. In this study, resistant *C. albicans* (CA10) cells were stained with CaspACE FITC-VAD-FMK to monitor the metacaspase response. As depicted in Figure 7, cells that were simultaneously exposed to HMH and FLC significant green fluorescence, indicating metacaspases activation. In the other three groups, only few fluorescent cells were observed.

## Discussion

*Candida albicans* is the most common human opportunistic fungal pathogen for immunocompromised patients. With the wide use of medical inbuilt catheters and the extensively abuse of conventional antifungal agents, fungal infections caused by *C. albicans*, especially resistant *C. albicans*, have become a severe clinical problem, warranting the need for newly improved antifungal strategies and therapies. As a research focus, combination therapies of antifungal drugs with non-antifungal agents have been proposed to treat drug-resistant *C. albicans* (Liu et al., 2014). To date, there has been no research on the combined antifungal effects of HMH with antifungal drugs. Nonetheless, HMH alone has been shown to exert antifungal effect on *C. albicans* (Nenaah, 2010). In this study, we evaluated the effects of azoles (FLC, ITR, and VRC) combined with HMH against *Candia* species *in vitro*. *G. mellonella* model was chosen to evaluate the effects of the drug combination *in vivo.* Furthermore, we sought to determine the possible synergistic mechanisms.

In the present study, we reported the MICs of drugs across all tested strains. The synergistic antifungal effects of HMH with FLC, ITR or VRC on resistant *C. albicans* strains were found. However, no synergy was seen with azole sensitive *C. albicans* strains nor with other *Candida* species. The results of MICs indicated that HMH and azoles can be combined as potential therapeutic protocols against resistant *C. albicans* infections.

The pathogenesis of *C. albicans* infection is mediated by a variety of virulence factors. Biofilm formation is a determinant of the cause of *C. albicans* infections (Uppuluri et al., 2010). Here, we found that biofilm pre-formed for 90 min was synergistically inhibited by the combination of HMH and azoles, thereby indicating the potential use of these combinations for the prevention or early treatment of biofilm-related *C. albicans* infections.

In recent years, a great similarity between results obtained from invertebrate models and mammalian models has been proven for drug efficacy evaluation (Cotter et al., 2000; Browne et al., 2013). To avoid the ethical issues associated with mammalian models and to reduce the cost of experiments, researchers have sought to develop invertebrate models to study microbial virulence (Cotter et al., 2000; Browne et al., 2013). Compared to mammalian models, *G. mellonella* model can be easily accessed, is large enough to be easily operated, and can survive at temperatures similar to mammalian hosts. Such advantages ultimately contribute to its wide acceptance as an invertebrate model for efficacy evaluation of antimicrobial agents (Brennan et al., 2002; Slater et al., 2011). In this study, the survival rate of infected larvae in the drug combination group was significantly improved compared to control group and drug monotherapy groups. Furthermore, the decreased fungal cells of tissues in the drug combination group depicted that the efficacy of FLC could be enhanced by HMH *in vivo*. The results showed that drug combination treatment was more efficacious than drug monotherapy for *C. albicans* growth inhibition. This is because the melanized nodules in larvae were less than that found in the drug monotherapy group. Therefore, the *in vivo* data further confirmed the *in vitro* antifungal effect of this drug combination and indicated the potential efficacy of this drug combination against resistant *C. albicans in vivo*.

Azole-resistant isolates commonly display reduced intracellular accumulation of azoles due to their overexpression of drug transport pumps. Functional activities of drug transport pumps have traditionally been assayed with R6G, a known fluorescent substrate of several drug transport pumps responsible for multidrug resistance in yeasts. Both R6G and azoles are the substrate of drug transport pumps. R6G was employed as the fluorescent alternate of azoles in this experiment (Holmes et al., 2012; De Cremer et al., 2015). For further explorations of mechanisms, we performed the R6G uptake and efflux assay to determine whether the synergism of HMH and azoles is relevant to the drug transporters. As a result, we detected no difference in the uptake of R6G between the HMH group and control group. However, HMH was found to significantly decrease the efflux of R6G, demonstrating that it reverses the resistance of resistant *C. albicans* to azoles by inhibiting the activity of drug transporters.

Besides biofilm, hyphal morphogenesis is considered to be one of the crucial virulence factors associated with the pathogenesis of *C. albicans* (Naglik et al., 2003; Dalle et al., 2010; Gow et al., 2011). The results showed that HMH combined with FLC has potential synergistic antifungal effects against the hyphae formation of resistant *C. albican*s. We suspected that hyphae formation, as one of the virulence factors, may be closely associated with drug resistance in *C. albicans*, and serve as a potent target for this drug combination against resistant *C. albicans*.

Calcium, as the second messenger in eukaryotic cells, plays a key role in overcoming drug resistance by fungus (Li and Sun, 2016). Hence, clarifying the calcium-related mechanisms of pathogenicity in *C. albicans* may contribute to the discovery of new targets for the treatment of resistant *C. albicans* infection. Our study found that HMH combined with FLC can increase [Ca^2+^]_i_ level of resistant *C. albicans*, revealing the important role of [Ca^2+^]_i_ in drug resistance of *C. albicans*.

ROS play an important physiological role in the survival of C. albicans and ROS generation is a momentous hallmark in fungal apoptosis (Madeo et al., 1999). As a primary cell death regulator, ROS can impact many crucial physiological pathways in fungus (Carmona-Gutierrez et al., 2010). In addition, studies have suggested that ROS production is involved in the antifungal mechanisms utilized by several antifungal agents on C. albicans (Sharma and Srivastava, 2014; Peralta et al., 2015; Lee and Lee, 2018). In the present study, resistant C. albicans exposed to the HMH and FLC drug combination produced a higher ROS level. This result suggested that ROS production could be involved in the antifungal mechanisms of this drug combination on resistant C. albicans. Metacaspases also play a central role in the apoptotic-signaling network of fungi. As metacaspase activity was significantly promoted by the drug combination in resistant C. albicans, the apoptotic process induced by this drug combination in resistant C. albicans was found to be mediated by a metacaspase-dependent apoptotic pathway.

**FIGURE 8:**
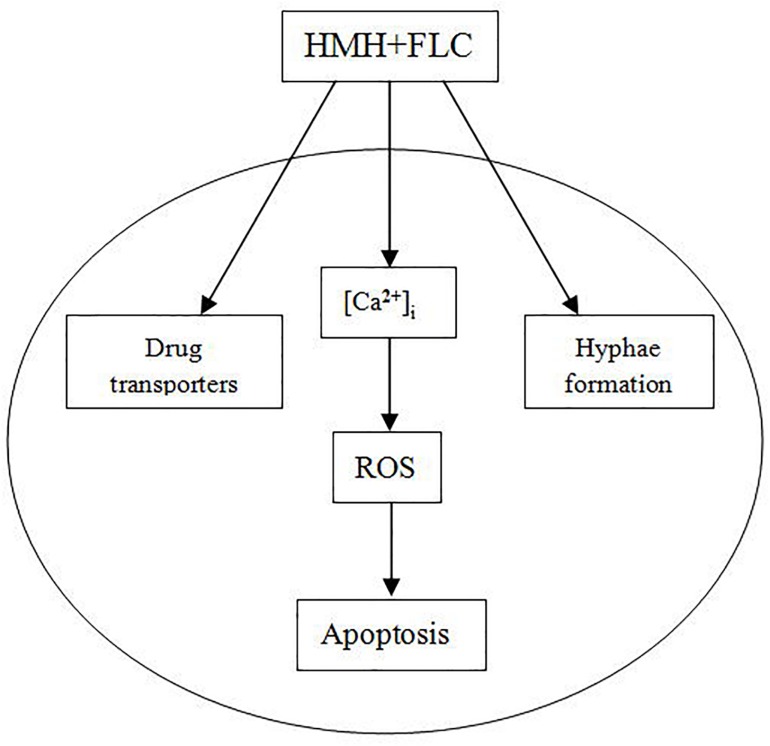
A model of the possible synergistic antifungal mechanisms.

A model for the antifungal mechanisms of HMH combined with FLC is provided in Figure 8. Such combination may exert synergistic antifungal effects on resistant *C. albicans* via three pathways (Figure 8). When resistant *C. albicans* cell is exposed to this drug combination, the activity of drug transporters and hyphae formation are both inhibited. Moreover, with stimulation by this combination, [Ca^2+^]_i_ level is disturbed, ROS increases, and finally, cell apoptosis occurs. Further future studies are needed or the inter-relations of the three pathways in Figure 8.

## Conclusion

Our results demonstrate the synergistic effects of HMH and azoles against resistant *C. albicans in vitro* (planktonic cells and biofilm in the early stage) and *in vivo*. To add, it suggests that the synergistic antifungal mechanisms may be involved in the inhibition of the efflux of azoles and hyphal growth, and the induction of apoptosis in resistant *C. albicans*.

Future studies are required on the mechanisms where by the synergistic antifungal effects of HMH and azoles occur.

## Data Availability Statement

The datasets analyzed in this manuscript are not publicly available. Requests to access the datasets should be directed to XL, lixiuyun666666@163.com.

## Author Contributions

XL and LH designed the experiments and conducted the *in vitro* and *in vivo* experiments. XL wrote the manuscript. LH corrected the manuscript. XW and YG conducted the experiments of synergistic mechanisms.

## Conflict of Interest

The authors declare that the research was conducted in the absence of any commercial or financial relationships that could be construed as a potential conflict of interest.
