# Supplementary material for: Synergistic Effects and Mechanisms of Combined Treatment With Harmine Hydrochloride and Azoles for Resistant Candida albicans
Source: Front Microbiol. 2019 Oct 15;10:2295. doi: 10.3389/fmicb.2019.02295 (PMC6843067; doi:10.3389/fmicb.2019.02295)
Supplement: Supplementary file 1 [file Table_1.docx]

**Table S1** A[verage](javascript:;) [value](javascript:;)s of cell growth rates for drug interactions of HMH and FLC against CA10

|  | | **FLC (μg/mL)** | | | | | | | | | | | |
| --- | --- | --- | --- | --- | --- | --- | --- | --- | --- | --- | --- | --- | --- |
|  |  | **0** | **0.125** | **0.25** | **0.5** | **1** | **2** | **4** | **8** | **16** | **32** | **64** | **空白** |
| **HMH (μg/mL)** | **512** | 15.63% | 8.80% | 8.14% | 8.05% | 7.14% | 6.14% | 5.81% | 5.64% | 4.14% | 3.89% | 2.65% | 0% |
|  | **256** | 53.71% | 29.70% | 8.14% | 7.85% | 7.34% | 8.21% | 6.54% | 5.37% | 5.96% | 5.22% | 5.38% | 0% |
|  | **128** | 78.04% | 31.61% | 10.03% | 9.28% | 9.70% | 8.04% | 7.45% | 7.29% | 6.93% | 6.28% | 6.37% | 0% |
|  | **64** | 81.61% | 31.12% | 11.03% | 10.70% | 10.12% | 8.54% | 8.20% | 7.45% | 8.88% | 8.46% | 6.30% | 0% |
|  | **32** | 88.25% | 49.92% | 12.92% | 11.41% | 10.55% | 12.81% | 11.10% | 7.36% | 7.95% | 8.95% | 8.95% | 0% |
|  | **16** | 97.17% | 55.74% | 15.16% | 14.16% | 11.54% | 8.30% | 8.58% | 10.02% | 9.44% | 10.86% | 10.12% | 0% |
|  | **8** | 99.92% | 67.99% | 16.22% | 15.81% | 15.64% | 14.12% | 13.87% | 14.12% | 12.54% | 12.87% | 13.21% | 0% |
|  | **0** | 100.00% | 89.32% | 85.91% | 85.08% | 77.70% | 70.13% | 59.65% | 51.41% | 49.50% | 46.09% | 40.70% | 0% |
